# Swedish dispatchers’ compliance with the American Heart Association performance goals for dispatch-assisted cardiopulmonary resuscitation and its association with survival in out-of-hospital cardiac arrest: A retrospective study

**DOI:** 10.1016/j.resplu.2021.100190

**Published:** 2021-12-24

**Authors:** Fredrik Byrsell, Andreas Claesson, Martin Jonsson, Mattias Ringh, Leif Svensson, Per Nordberg, Sune Forsberg, Jacob Hollenberg, Anette Nord

**Affiliations:** a Department of Clinical Science and Education, Centre for Resuscitation Science, Karolinska Institutet, Södersjukhuset, Stockholm, Sweden; b Department of Medicine, Solna Karolinska Institutet, Stockholm, Sweden; c SOS Alarm AB, Stockholm, Sweden

**Keywords:** Out-of-hospital cardiac arrest (OHCA), Emergency calls, Emergency medical dispatch centre, Dispatcher, Cardiopulmonary resuscitation, Emergency medical services, Resuscitation, DA-CPR

## Abstract

**Aim:**

We aimed 1) to investigate how Swedish dispatchers perform during emergency calls in accordance with the American Heart Association (AHA) goals for dispatcher-assisted cardiopulmonary resuscitation (DA-CPR), 2) calculate the potential impact on 30-day survival.

**Methods:**

This observational study includes a random sample of 1000 out-of-hospital cardiac arrest (OHCA) emergency ambulance calls during 2018 in Sweden. Voice logs were audited to evaluate dispatchers’ handling of emergency calls according to the AHA performance goals. Number of possible additional survivors was estimated assuming the timeframes of the AHA performance goals was achieved.

**Results:**

A total of 936 cases were included. An OHCA was recognized by a dispatcher in 79% (AHA goal 75%). In recognizable OHCA, dispatchers recognized 85% (AHA goal 95%). Dispatch-directed compressions were given in 61% (AHA goal 75%). Median time to OHCA recognition was 113 s [interquartile range (IQR), 62, 204 s] (AHA goal < 60 s). The first dispatch-directed compression was performed at a median time of 240 s [IQR, 176, 332 s] (AHA goal < 90 s). If eligible patients receive dispatch-directed compressions within the AHA 90 s goal, 73 additional lives may be saved; if all cases are recognized within the AHA 60 s goal, 25 additional lives may be saved.

**Conclusions:**

The AHA policy statement serves as a benchmark for all emergency medical dispatch centres (EMDC). Additional effort is needed at Swedish EMDC to achieve AHA goals for DA-CPR. Our study suggests that if EMDC further optimize handling of OHCA calls in accordance with AHA goals, many more lives may be saved.

## Introduction

Recognition of out-of-hospital cardiac arrest (OHCA) in emergency calls by dispatchers, including telecommunicators, is time critical. Reducing times to OHCA recognition, start of dispatch-assisted cardiopulmonary resuscitation (DA-CPR) and dispatch of emergency medical services (EMS) are essential to increasing survival after OHCA.1., 2., 3., 4., 5., 6. In Sweden, about 6000 cases of OHCA are reported to the Swedish Register of Cardiopulmonary Resuscitation (SRCR) annually, and the 30-day survival rate is about 11%.^7^ Witnessed OHCA, agonal breathing and patients manifesting seizures have been shown to complicate recognition of an OHCA.8., 9., 10., 11., 12. The proportion of recognized OHCA varies greatly between emergency medical dispatch centres (EMDC) and countries.^9^ One study showed recognition rates from EMDC of 71% in Copenhagen, Denmark, 96% in Oslo, Norway and 83% in Stockholm, Sweden.^13^

**Fig. 1:**
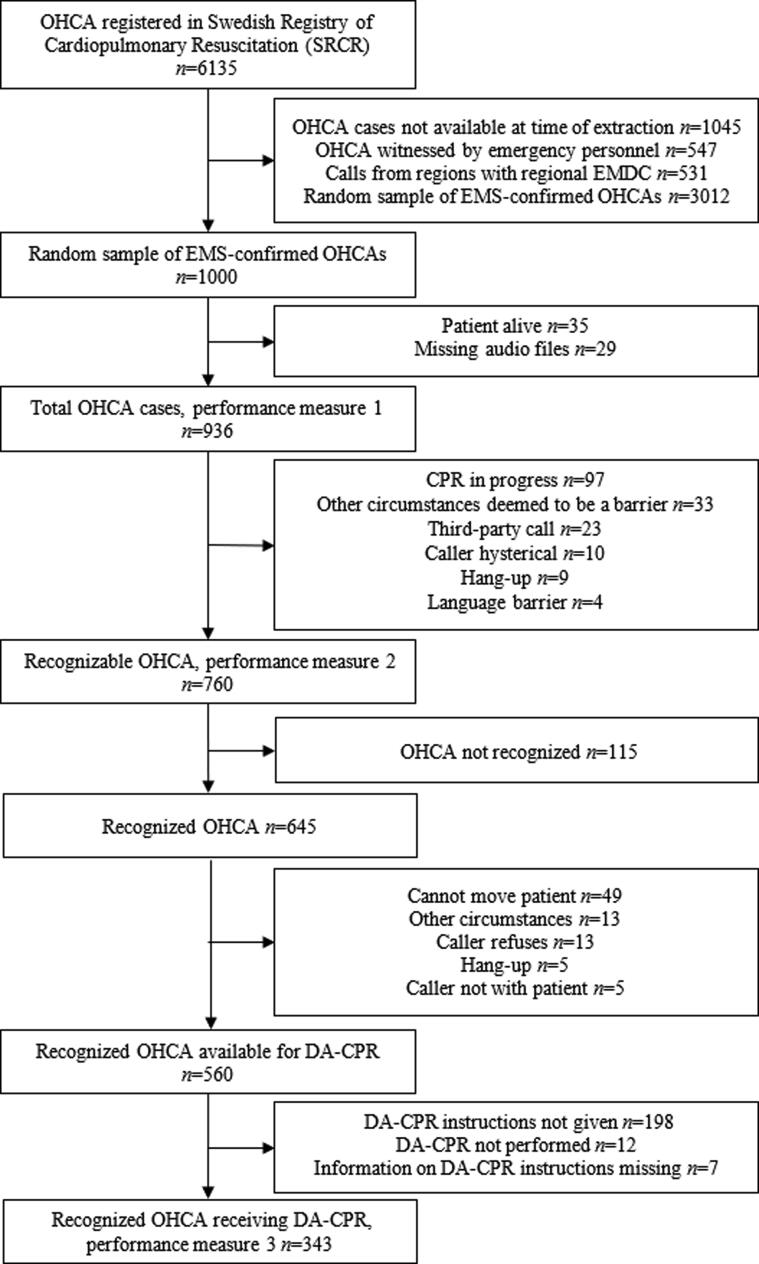
Flowchart of the study.

The American Heart Association’s (AHA) policy statement describes clinical performance goals for OHCA call handling:^14^ (1) percentage of total OHCA cases correctly recognized by dispatchers, (2) percentage of OHCA cases correctly recognized by dispatchers that were recognizable (i.e. excluding cases deemed unidentifiable according to AHA exclusion criteria such as third-party call, hang up, hysteria, language barrier etc, see Fig. 1), (3) percentage of dispatcher-recognized OHCA receiving DA-CPR, (4) median time between receiving emergency call and OHCA recognition and dispatch of first unit, (5) median time between emergency call and first DA-CPR-directed compression. The AHA performance goals for DA-CPR can be used to evaluate dispatchers’ performance and as feedback for quality improvement (QI) purposes, such as self-audit of emergency calls.

The primary aim of this study was to describe medical dispatchers’ performance in Swedish EMDC in OHCA emergency calls in accordance with the AHA performance goals for DA-CPR. Our secondary aim was to assess the probability of 30-day survival related to time to recognition and time to first chest compression delivered by bystander and to calculate the number of additional survivors if all patients received DA-CPR within 90 s.

## Methods

### Study design

This is an observational retrospective study using a random sample of register-based data and data from a manual audit of emergency calls for OHCA treated by the EMS in Sweden in 2018. Ethical approval was received from the Swedish ethical review authority (DNR: 2019–01998).

### Swedish register for cardiopulmonary resuscitation

The SRCR is a national quality register. All Swedish EMS organizations report attempted resuscitations. The OHCA are reported to the registry when bystanders or EMS have initiated resuscitation efforts with cardiopulmonary resuscitation (CPR) and/or defibrillation. The SRCR follows the Utstein template for OHCA registration.^15^ An extensive description of the SRCR has been described previously.16., 17.

### Swedish dispatch organization

The Swedish national emergency number is run by SOS Alarm AB which is publicly owned through the Swedish government and Sweden's municipalities and regions. Dialling the emergency number directs the call to one of 15 EMDC. In Sweden, all emergency calls are answered directly by medical dispatchers at EMDC and an ambulance dispatcher is responsible for the dispatch of EMS. Medical dispatchers handle emergency calls from start to finish, with the exception of three regions where the call is redirected by the dispatcher to regional EMDC run by regional EMS organizations.

To become a medical dispatcher at SOS Alarm AB, trainees must undergo 14 weeks of education. A re-certification test is performed annually. The dispatchers are supported in their medical decision-making process by a criteria-based medical index.^18^ Criteria-based medical index means that the index guides dispatchers based on the described symptoms and provide direction and assistance in determining a suitable priority.^19^ Continuous QI regarding OHCA calls is not regularly evaluated in the manner proposed by the AHA.^14^

### Study population

A total of 6135 cases of OHCA were registered in the SRCR by the EMS in 2018. After excluding OHCA cases not available at the time of extraction, cases handled by care providers from regions where SOS Alarm AB does not assess the medical urgency and cases witnessed by EMS personnel, a random non-stratified sample (*n* = 1000) was selected for inclusion in the study using Stata statistical software.^20^ We estimated that 1000 emergency calls were sufficient to reach data saturation as well as a manageable sample size to audit. Further exclusions were made to adhere to exclusions described by the AHA performance measures.^14^

### Manual audit

A manual audit in terms of listening to all OHCA call voice logs (call recordings) was performed in order to more precisely evaluate dispatchers performance according to the AHA performance measures. A modified version of the Cardiac Arrest Registry to Enhance Survival (CARES) dispatcher-assisted CPR data dictionary was used to extract data from the voice logs (Supplementary File 1).^21^ The manual audit was conducted by two investigators. An interrater reliability analysis was performed to evaluate consistency in assessment between investigators. The intraclass correlation analysis showed values > 0.9, indicating high reliability for the time variables related to the study endpoints. Information regarding patient characteristics was extracted from the SRCR. The definition of “time to recognized OHCA” by the dispatcher was when the dispatcher verbally declared the need for CPR or the presence of an OHCA. Elapsed time to OHCA recognition was measured from the timestamp when the emergency call was answered until OHCA recognition was achieved. Time to first chest compression was the time elapsed from the start of the call to the moment when the caller or rescuer performed the first chest compression.20., 21.

### Outcome measures

The endpoints in this study are as defined by the five AHA policy statements for DA-CPR.^14^

1. Percentage of total OHCA cases correctly recognized by dispatchers: performance goal 75%.
2. Percentage of OHCA cases correctly recognized by dispatchers that were recognizable (i.e. excluding cases deemed unidentifiable according to AHA exclusion criteria such as third-party call, hang up, hysteria, language barrier etc, see Fig. 1): performance goal 95%.
3. Percentage of dispatcher-recognized OHCA receiving DA-CPR: performance goal 75%.
4. Median time between the emergency call and OHCA recognition and dispatch of first unit: high-performance system < 60 s, minimal acceptable standard < 90 s.
5. Median time between emergency call and first DA-CPR-directed compression: high-performance system < 90 s, minimal acceptable standard < 150 s.

In addition, the probability of 30-day survival related to time to recognition, time to first chest compression and an estimation of additional survivors if all victims were recognized within 60 s, dispatch of first unit within 60 s or received DA-CPR within 90 s was calculated by applying an estimation calculation described previously.^22^

### Statistical analysis

Descriptive data are presented as the proportion (%) or median (interquartile range). The probability of 30-day survival in relation to time to recognition and time to chest compressions is illustrated through restricted cubic splines. Estimations on additional survivors depending on overall survival (OS) rates in different timeframes were calculated using a mathematical formula previous used and described by Strömsöe et al.^22^ The overall survival rate of the sample that met the time target is subtracted with the overall survival rate of the sample that did not meet the time target, and is then multiplied by the number of patients not meeting the time target. Analyses were performed using IBM SPSS version 27 and R version 3.6.1.

## Results

Of the 6135 cases of OHCA registered in the SRCR in 2018, subsequent exclusion of cases not available at the time of extraction (*n* = 1045), cases witnessed by the EMS (*n* = 547), cases from regions with regional EMS organizations (*n* = 531) resulted in 4012 cases eligible for inclusion. A random sample (*n* = 1000) was extracted for inclusion in the study (Fig. 1).

### Performance goal 1: Percentage of total OHCA cases correctly recognized by dispatchers

After excluding emergency calls involving patients who were alive during the call (*n* = 35) and cases with missing audio files (*n* = 29), 936 OHCA cases were included (Fig. 1). An OHCA was recognized by a dispatcher in 79% (*n* = 742) (AHA performance goal 75%). The OHCA call characteristics are shown in Table 1.

**Table 1:** Characteristics of Out-of-Hospital Cardiac Arrest Calls.

	All OHCAs (*n* = 936)	OHCA recognized by dispatcher (*n* = 645)	OHCA not recognized by dispatcher (n = 115)
*Patient age (years), median [IQR]	72 [61,81]	72 [63,81]	75 [65,82]
*Patient female	318 (34)	219 (34)	43 (37)
Missing	7 (1)	5 (1)	0 (0)
Caller female	550 (59)	398 (62)	65 (57)
Missing	30 (3)	1 (<1)	0 (0)
Caller alone	348 (37)	268 (42)	40 (35)
Missing	45 (5)	4 (1)	7 (6)
Caller health care professional	259 (28)	179 (28)	38 (33)
Missing	34 (4)	1 (<1)	0 (0)
Caller relationship with patient			
Knows the victim	780 (83)	566 (88)	94 (82)
Unknown victim	121 (13)	76 (12)	21 (18)
Missing	35 (4)	3 (<1)	0 (0)
*OHCA location			
Residential	677 (72)	495 (77)	80 (70)
Public	176 (19)	106 (16)	24 (21)
Other	79 (8)	41 (6)	10 (9)
Missing	4 (<1)	3 (<1)	1 (1)
*OHCA witnessed			
Yes	531 (57)	370 (57)	73 (63)
No	376 (40)	254 (39)	35 (30)
Missing	29 (3)	21 (3)	7 (6)
CPR in progress	97 (10)	0(0)	0(0)
Patient consciousness addressed			
Yes	810 (87)	580 (90)	106 (92)
No	80 (9)	61 (9)	8 (7)
Missing	46 (5)	4 (1)	1 (1)
Patient breathing addressed			
Yes	872 (93)	632 (98)	109 (95)
No	21 (2)	12 (2)	5 (4)
Missing	43 (5)	1 (<1)	1 (1)
*Cause of OHCA			
Cardiac disease	512 (55)	370 (57)	60 (52)
Overdose	21 (2)	12 (2)	4 (3)
Trauma	19 (2)	8 (1)	5 (4)
Respiratory disease	46 (5)	33 (5)	6 (5)
Asphyxia	28 (3)	23 (3)	2 (2)
Suicide	38 (4)	14 (2)	4 (3)
Submersion	19 (2)	3 (1)	2 (2)
SIDS	7 (1)	5 (1)	0 (0)
Other	189 (20)	133 (21)	24 (21)
Missing	57 (6)	44 (7)	8 (7)
*Shockable rhythm	170 (18)	121 (19)	16 (14)
Missing	12 (1)	5 (1)	2 (2)
AED addressed	68 (7)	50 (8)	0 (0)
Missing	82 (9)	15 (2)	14 (12)
AED shock before EMS	9 (1)	8 (1)	0(0)
Missing	83 (9)	15 (2)	14 (12)
Call continued until EMS arrival			
Yes	488 (52)	423 (66)	17 (15)
No	347 (37)	198 (31)	79 (69)
Missing	101 (11)	24 (4)	19 (17)
*30-day survival	66 (7)	45 (7)	8 (7)
Missing	47 (5)	30 (5)	8 (7)

Values are number (%) except where indicated otherwise. Percentages are rounded to the nearest integer.

AED, automated external defibrillator; CPR, cardiopulmonary resuscitation; EMS, emergency medical services; IQR, interquartile range; OHCA, out-of-hospital cardiac arrest; SIDS, sudden infant death syndrome. Data retrieved from the SRCR are marked *

### Performance goal 2: Percentage of recognizable OHCA cases correctly recognized by dispatchers

After excluding emergency calls in accordance with the AHA criteria involving CPR in progress (*n* = 97), third-party call (*n* = 23), caller hysterical (*n* = 10), caller hang-ups (*n* = 9), language barrier (*n* = 4) and other circumstances deemed to be a barrier (*n* = 33), 760 recognizable OHCA remained (Fig. 1). Of these, dispatchers recognized 85% (*n* = 645) (AHA performance goal 95%). The call characteristics are shown in Table 1.

### Performance goal 3: Percentage of Dispatcher-Recognized OHCA receiving DA-CPR

After excluding cases in accordance with the AHA criteria where the caller cannot move patient (*n* = 49), caller refuses to perform CPR (*n* = 13), caller hangs up (*n* = 5), caller is not with the patient (*n* = 5) and other circumstances deemed as barrier (*n* = 13), a total of 560 OHCA were available for DA-CPR (Fig. 1). Sixty-one percent of OHCA received DA-CPR-directed compressions (AHA performance goal 75%).

Overall, of the OHCA available for DA-CPR (*n* = 560), 90% (*n* = 504) received chest compressions during the call (i.e. DA-CPR chest compressions or spontaneously bystander-initiated chest compressions).

### Performance goal 4: Median time between receiving the emergency call and OHCA recognition and dispatch of the first unit

Median time to OHCA recognition by dispatchers (*n* = 528) was 113 s [interquartile range (IQR), 62, 204 s] (AHA < 60 s high-performance system goal, and <90 s minimal acceptable standard). Twenty percent (*n* = 150) were recognized as OHCA by dispatchers within 60 s and 35% (*n* = 267) were recognized within 90 s (Table 2).

**Table 2:** Dispatcher performance in emergency calls for Out-of-Hospital Cardiac Arrest.

Recognizable OHCA (*n* = 760)	Overall
OHCA recognized	645 (85)
Time to recognition (seconds), median [IQR]	113 [62, 204]
Recognition < 60 s	150 (20)
Recognition < 90 s	267 (35)
Time to recognition missing	117 (15)
Time to EMS dispatch (seconds), median [IQR]	88 [64, 131]
Dispatch < 60 s	154 (20)
Dispatch < 90 s	386 (51)
**Recognized OHCAs available for DA-CPR (*n* = 560)**	
Any chest compression performed	504 (90)
Time to first chest compression (seconds), median [IQR]	214 [146, 315]
Chest compression < 90 s	31 (6)
Chest compression < 150 s	109 (19)
No time established (missing)	136 (24)
***DA-CPR directed chest compression***	
DA-CPR chest compression performed	343 (61)
Time to CPR instructions (seconds), median [IQR]	201 [137, 300]
Missing	7 (1)
Time to first DA-CPR chest compression (seconds), median [IQR]	240 [176, 332]
Missing	40 (7)
DA-CPR directed compressions < 90	9 (2)
DA-CPR directed compressions < 150	52 (9)

Values are number (%) except where indicated otherwise.

DA-CPR, dispatcher-assisted-cardiopulmonary resuscitation; EMS, emergency medical services; IQR, interquartile range; OHCA, out-of-hospital cardiac arrest.

Median time to dispatch of the first unit in all cases of OHCA (*n* = 935) was 87 s [IQR, 63, 132 s]. Median time to dispatch of the first unit in recognizable OHCA (*n* = 760) was 88 s [IQR, 64, 131 s]; 20% (*n* = 154) were dispatched within the AHA high-performance goal of <60 s and 51% (*n* = 386) were dispatched within the AHA minimal acceptable standard of <90 s (Table 2).

### Performance goal 5: Median time between emergency call and first DA-CPR-directed chest compression

The first DA-CPR-directed compression (*n* = 303) was performed at a median time of 240 s [IQR, 176, 332 s]. A total of 6% (*n* = 31) received any chest compression within AHA high-performance goal of <90 s and 19% (*n* = 109) within the AHA minimal acceptable standard of <150 s (Table 2).

### 30-Day estimated survival

Overall 30-day survival in all cases of OHCA (*n* = 936) was 7% (*n* = 66). If time to recognition >60 s was reduced to <60 s, an additional 25 lives could be saved (overall survival rate at >60 s less overall survival rate at <60 s: 10.52%−7.056% = 3.464% × 727 = 25 lives).

If the time to dispatch of the first unit for all OHCA cases was reduced from >60 s to <60 s, an additional 10 lives could be saved (overall survival rate at >60 s less overall survival rate at < 60 s: 8.556%−7.142% = 1.414% × 749 = 10 lives).

If the time to chest compression for all OHCA cases available for DA-CPR was reduced from >90 s to <90 s, an additional 73 lives could be saved (overall survival rate at >90 s less overall survival rate at <90 s: 20%−6.15%=13.85%×530 = 73 lives).

**Fig. 2:**
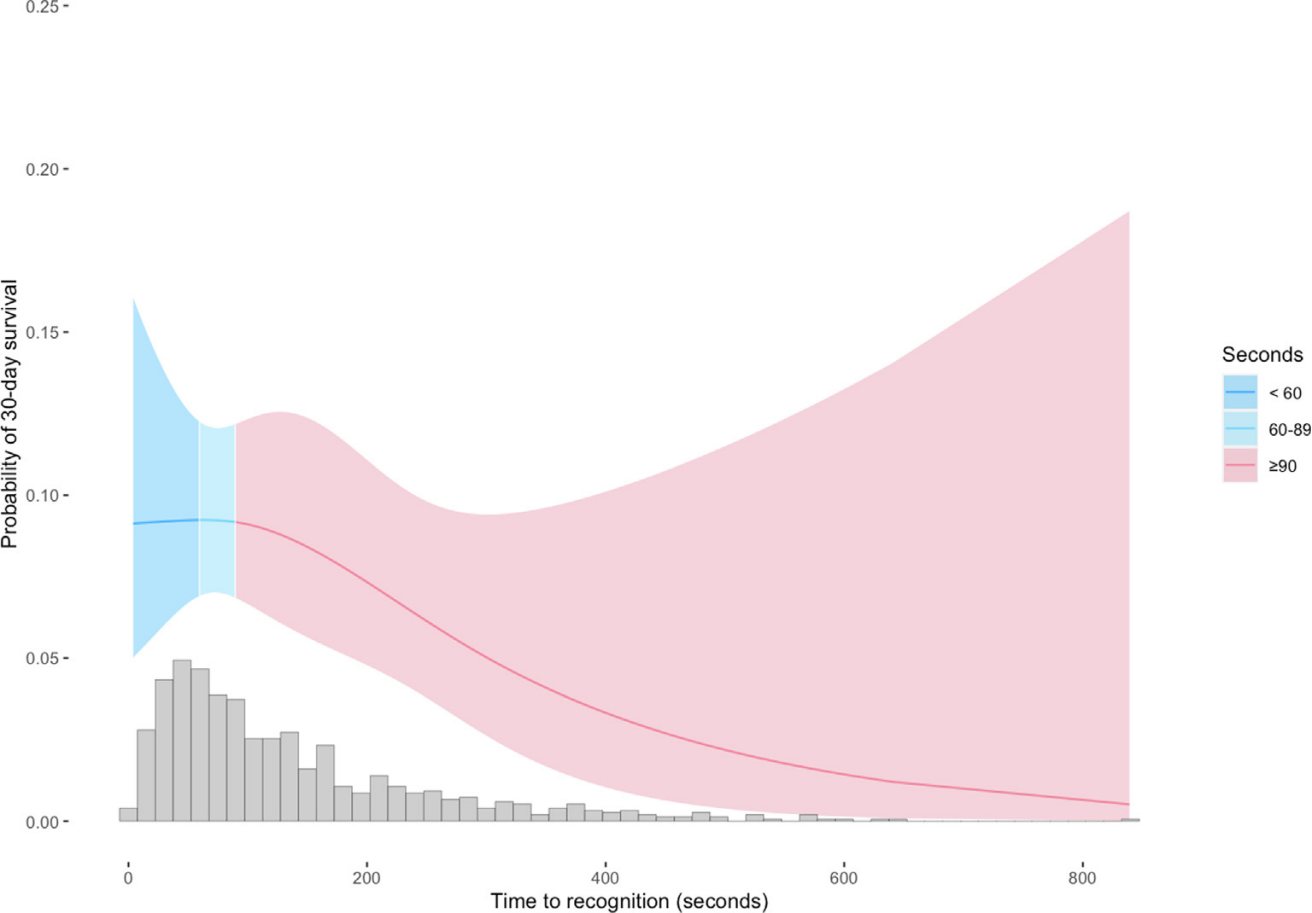
Probability of 30-day survival depending on time to OHCA recognition (n = 936).

**Fig. 3:**
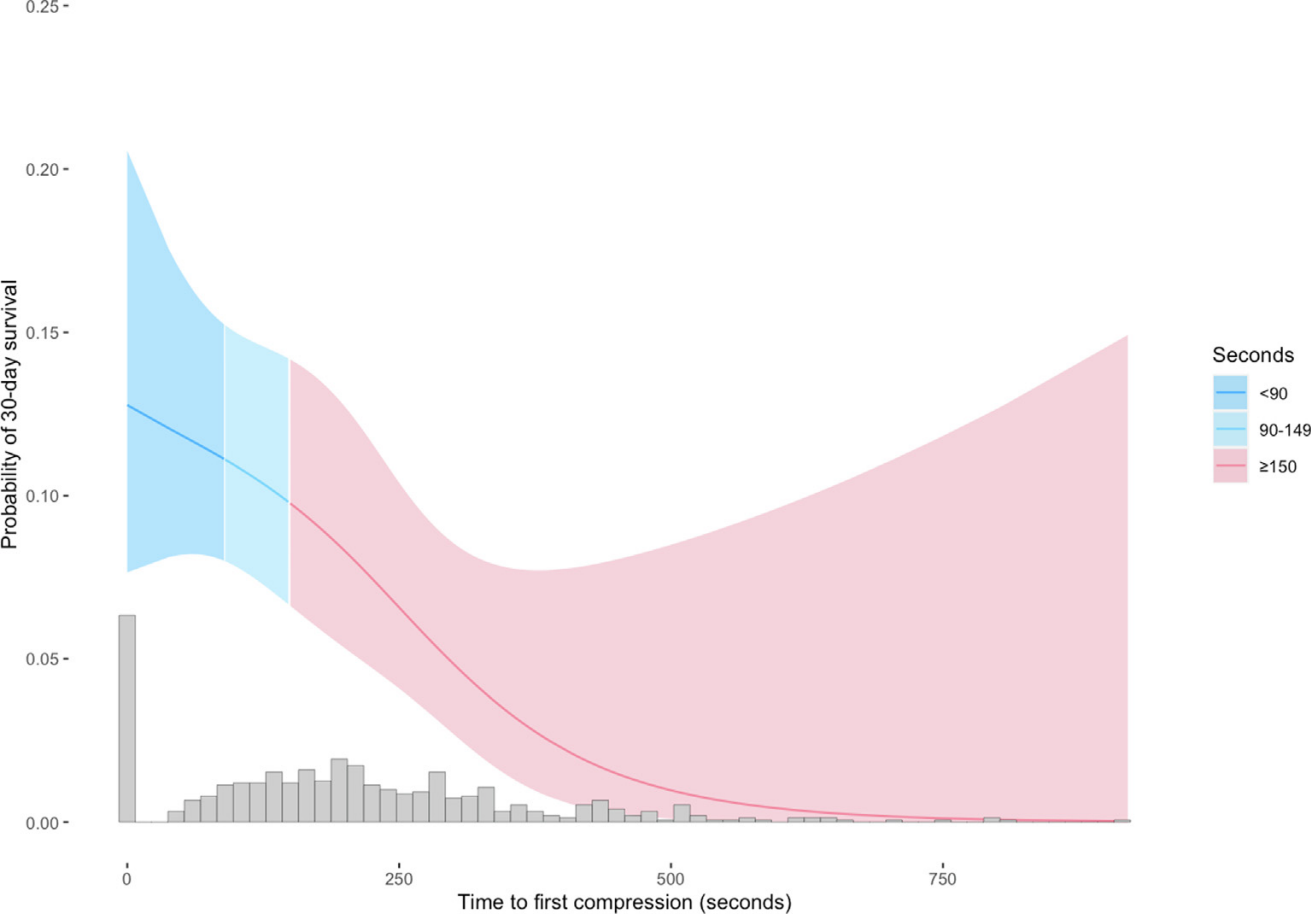
Probability of 30-day survival depending on time to start of chest compressions (n = 936).

**Fig. 4:**
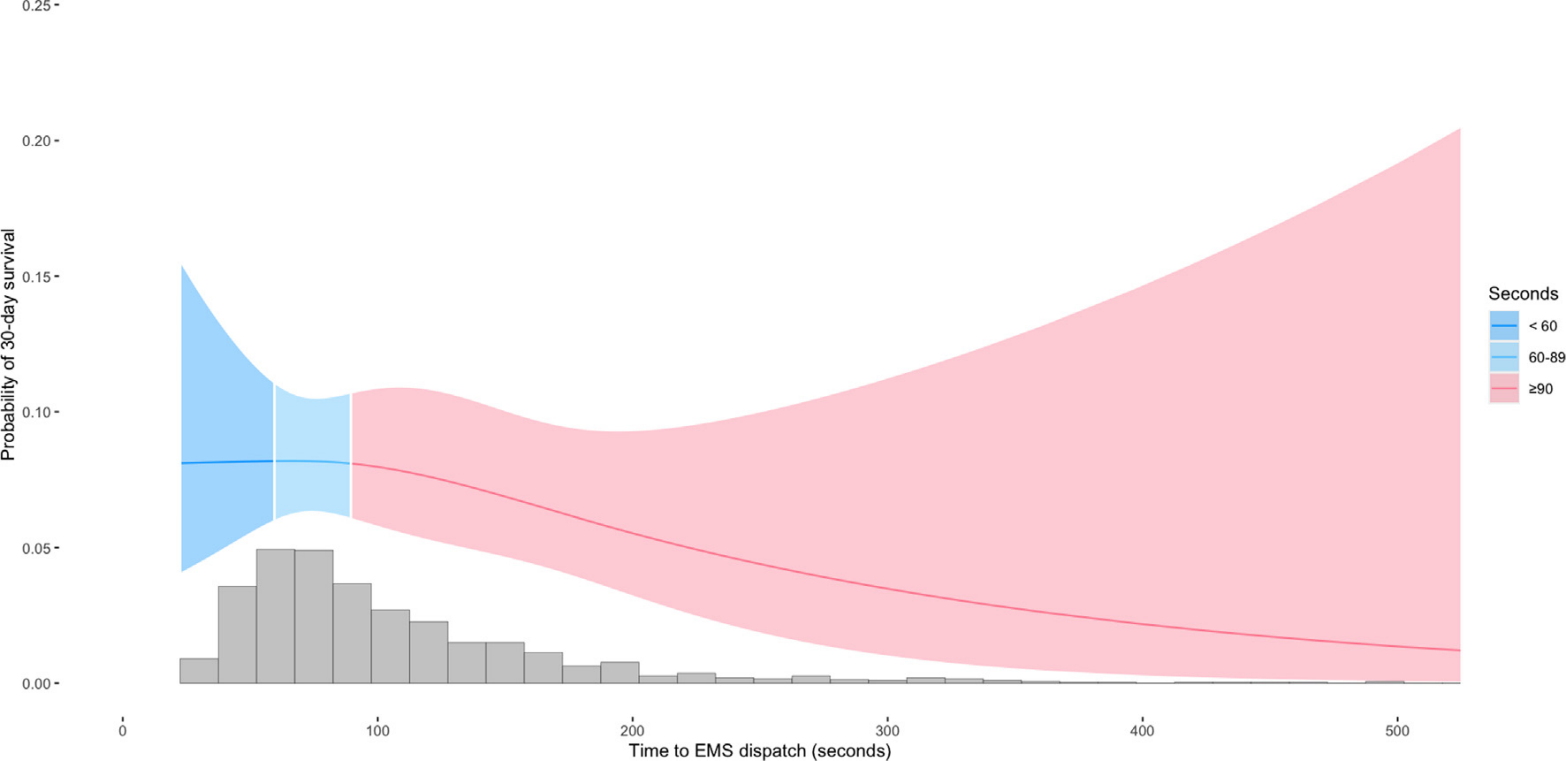
Probability of 30-day survival depending on time to EMS dispatch (n = 936).

Fig. 2, Fig. 3, Fig. 4 are restricted cubic splines illustrating the probability of 30-day survival depending on time to recognition, time to first chest compression and time to EMS dispatch.

## Discussion

The present study describes the performance of Swedish EMDC on emergency calls involving OHCA in accordance with the AHA policy statement for DA-CPR.^14^ Our main findings show that additional effort is needed to achieve the performance goals set by the AHA. Our calculated estimations indicate that the probability of 30-day survival may depend on time to recognition, time to dispatch of EMS and time to first chest compressions.

The AHA’s first performance measure is the percentage of OHCA cases correctly recognized by dispatchers. Our study shows that an OHCA was recognized by a dispatcher in 79% of cases, exceeding the AHA 75% performance goal. However, 21% are missed, i.e. more than 1200 cases of OHCA per year in Sweden. Early recognition and start of CPR before EMS arrival is essential for survival.8., 14., 23., 24. Additional efforts are needed to save more lives.^25^

The second performance measure is percentage of OHCA cases correctly recognized by dispatchers that were recognizable (i.e. excluding cases deemed unidentifiable according to AHA exclusion criteria such as third-party call, hang up, hysteria, language barrier etc, see Fig. 1). The AHA performance goal of 95% was not achieved; only 85% of OHCA were recognized. One reason could be witnessed OHCA when patients present agonal breathing patterns, and these are misinterpreted as normal breathing.^26^ In 2018, SOS Alarm AB had no mandatory routine to ask for abnormal breathing, only if the victim was breathing. About 50% of OHCA patients are breathing abnormally during the first minutes.14., 27., 28. Studies have shown that agonal breathing has a negative influence on dispatchers’ inclination to suspect an OHCA.8., 9., 10., 12.

The third performance measure is percentage of dispatcher-recognized OHCA receiving DA-CPR. Our study shows that 61% of OHCA patients received DA-CPR, which does not meet the AHA performance goal of 75%. However, of the OHCA available for DA-CPR, 90% received chest compressions. Dispatchers might not consider providing CPR instructions if callers are trained in CPR or are health care professionals. Despite a caller’s previous CPR knowledge, dispatchers should provide CPR instructions, conduct quality control and support callers, to ensure that CPR is performed correctly.^14^

The AHA’s fourth performance measure is median time between the emergency call and OHCA recognition (high-performance system < 60 s, minimal acceptable standard < 90 s). Our study shows a need to improve time to recognition since median time to OHCA recognition by dispatchers was 113 s. Shortening time to CPR and EMS response is essential to increase survival in patients with OHCA.2., 24. When consciousness and breathing can be assessed, the “no, no, go” process can facilitate a 92% recognition rate. This is a short and straightforward process where the dispatchers asks if the patient is conscious and if the patient is breathing normally. If the reply is “no” on both questions the dispatcher should “go” on and suspect an OHCA, provide DA-CPR instructions and dispatch EMS.^14^ The “no, no, go” process is not established in Swedish EMDC. For this random sample, we estimated that a further 25 lives could be saved if all OHCA were recognized within 60 s. Based on all OHCA in Sweden (*n* = 6135), and assuming similar patient characteristics, this means more than 150 additional lives can be saved annually.

The median time to dispatch of EMS was 87 s in all OHCA, which is in accordance with AHA’s minimal acceptable standard. We estimated that a further 10 lives could be saved if time to dispatch of EMS was within 60 s. Based on all OHCA in Sweden, and assuming similar patient characteristics, this means an additional 60 lives can be saved annually.

The AHA’s fifth performance measure is the median time between the emergency call and the first DA-CPR-directed compression (high-performance system < 90 s and minimal acceptable standard < 150 s). Although cases with aggravating circumstances were excluded, the median time to first DA-CPR-performed chest compression of 240 s indicates that efforts are needed to shorten the time. Callers often have difficulty getting the patient into the correct position, delaying the start of CPR; this affects the results and occurs in 20% of OHCA calls available for DA-CPR. If the rescuer cannot move the victim to a hard surface, it is better to start CPR in situ. Effective chest compression depths can be achieved even on a soft surface if the rescuer increases the overall compression depth to compensate for compression of the soft surface.^25^ We estimated that a further 73 lives could be saved if all patients available for DA-CPR received chest compressions within 90 s. Based on all OHCA cases in Sweden with a survival rate of about 11% (*n* = 626), and assuming similar patient characteristics, more than 438 additional lives could be saved annually.

### Strategies for Early recognition of OHCA and start of DA-CPR

An important organizational commitment is to audit and measure performance of OHCA calls for QI purposes in accordance with the AHA policy statement. Effective QI that results in a blame-free effort to identify barriers and educational needs improves overall survival.14., 29. Continuous and effective QI of OHCA calls is not implemented at SOS Alarm AB.

Several obstacles (hysterical callers, hang-ups, etc.) are difficult to overcome. Therefore, there is an urgent need to identify new and effective measures to overcome these barriers in the future and to shorten the time for OHCA recognition and start of treatment.^30^ Quality improvement programmes and how dispatchers communicate with callers can affect performance in OHCA calls.31., 32. Hardeland et al.^33^ have shown that education, targeted simulations as well as feedback on OHCA recognition during emergency calls improved dispatchers’ ability to recognize OHCA (89–95%) and delayed OHCA recognition was reduced (21–6%).

European Resuscitation Council guidelines 2021 specify that EMDC systems should consider new technology, such as artificial intelligence, to assist in recognizing OHCA. Machine learning (ML) trained to recognize OHCA in emergency calls has been proposed as a supportive tool for dispatchers in emergency calls. In retrospective studies, ML showed higher sensitivity^20^ and specificity compared with dispatchers in recognizing OHCA.^34^ However, a randomized controlled trial showed no improvement in dispatchers’ ability to identify OHCA when supported by ML, even though ML surpassed human recognition. One reason may be dispatchers’ lack of compliance with the ML advice.^35^ Combining systematic feedback on OHCA call performance for QI and consistent education, training, and simulation for dispatchers, in combination with a ML supportive tool, could improve recognition rates and time to recognition. Other future technical tools could include smartphone applications and smart speakers to facilitate OHCA recognition and smartwatches or video communication with smartphones to check the quality of DA-CPR provided.29., 25. These strategies are complementary, and no single approach should be considered as an independent endeavour.^14^ Further research is needed to investigate how dispatchers can be optimally supported or the use of technical tools during emergency calls.^36^

### Limitations

First, in terms of identifying the time to OHCA recognition via the voice logs, dispatchers could have recognized an OHCA earlier, but did not verbally communicate the need for CPR due to circumstances at the scene. Second, in some countries, the emergency call is transferred from a public safety answering point; in Sweden, all calls are handled by the dispatcher directly. Thus, it is difficult to compare the time to recognition between studies. Third, noting the time of first chest compression proved difficult in 24% of the calls available for DA-CPR, making the result of time to chest compression uncertain. By excluding some calls according to the AHA policy statement criteria (Fig. 1), there is a risk that the overall results are false positives. For example, 8% (*n* = 49) of the recognized OHCA were excluded because the caller was unable to get the patient into the appropriate position for CPR. Maybe the dispatcher, with other instructions, could prompt the caller to start CPR in situ, instead of accepting that the caller cannot get the patient onto a hard surface. Fourth, the sample is limited (1000 OHCA), and we cannot exclude the possibility that other predictors for survival (e.g. initial rhythm or age) affected the results.^37^ Thus, our calculations may be overly optimistic. However, the strong association between delay to recognition and DA-CPR/chest compressions and overall survival in OHCA has been shown previously.22., 23., 38.

## Conclusion

The AHA policy statement serves as a benchmark for all emergency medical dispatch centres (EMDC). Additional effort is needed at Swedish EMDC to achieve AHA goals for DA-CPR. Our study suggests that if EMDC further optimize handling of OHCA calls in accordance with AHA goals, many more lives may be saved.

## Funding

Funding was received from Region Stocholm, ALF project, which had no role in the design, analysis or writing of the article.

## CRediT authorship contribution statement

**Fredrik Byrsell:** Conceptualization, Methodology, Data curation, Formal analysis, Writing – original draft, Writing – review & editing. **Andreas Claesson:** Conceptualization, Methodology, Writing – review & editing. **Martin Jonsson:** Formal analysis, Writing – review & editing. **Mattias Ringh:** Conceptualization, Methodology, Writing – review & editing. **Leif Svensson:** Conceptualization, Methodology, Writing – review & editing. **Per Nordberg:** Writing – review & editing. **Sune Forsberg:** Writing – review & editing. **Jacob Hollenberg:** Conceptualization, Writing – review & editing. **Anette Nord:** Conceptualization, Methodology, Formal analysis, Writing – review & editing.

## Declaration of Competing Interest

The authors declare the following financial interests/personal relationships which may be considered as potential competing interests: F. Byrsell is an employee at SOS Alarm AB. None of the other authors have any conflicts of interest to declare.

## Appendix A Supplementary material

Supplementary Data 1 Instructions for audit.
