# Supplementary material for: Swedish dispatchers’ compliance with the American Heart Association performance goals for dispatch-assisted cardiopulmonary resuscitation and its association with survival in out-of-hospital cardiac arrest: A retrospective study
Source: Resusc Plus. 2021 Dec 24;9:100190. doi: 10.1016/j.resplu.2021.100190 (PMC9076962; doi:10.1016/j.resplu.2021.100190)
Supplement: Supplementary Data 1 — Instructions for audit. [file mmc1.docx]

**Instruction template for audit of cardiac arrest calls**

English version (2020-12-08)

Contents

1. Call assessment necessary 4

2. Audio file available 4

3. Country 4

4. Data collector initials 4

5. Filename 4

6. Time of dispatcher addressing caller (MM:SS) 4

7. CPR already in progress? 4

8. Consciousness addressed? 5

9. Patient is conscious? 5

10. Breathing addressed? 6

11. Abnormal breathing adressed 6

12. Patient is breathing normally? 7

13. Did dispatcher recognise need for CPR? 8

14. Delayed recognition due to deviation from cardiac arrest protocol 8

15. Time of recognition (MM:SS) 9

16. BLS competence addressed? 10

16.1 Is the collapse witnessed? 10

16.2 Bystander has previous CPR training? 11

16.3 Age above 18 years? 11

16.4 Probable cause of CA of medical origin and not in combination with trauma, drowning, suffocation, intoxication or apparent pregnancy? 11

17. CPR instructions started? 12

18. Is the dispatcher assertive or passive when providing CPR instructions? 12

19. Quality assessment 13

20. Encouraging/motivating techniques in use 13

21. Type of CPR 13

22. Time of chest compression instructions started (MM:SS) 14

23. Chest compressions started? 14

24. Time of first chest compression (MM:SS) 15

25. Was an AED addressed 16

26. Was an AED connected to the patient? 16

27. Did the AED deliver a shock to the patient? 16

28. Barriers delaying or hindering CPR? 16

29. Was CA witnessed by another person? 18

30. Call continued until EMS arrival 18

31. Caller gender 19

32. Caller relation to patient 19

33. Healthcare professional 19

34. Was caller alone at time of call? 19

**Abbreviations**

AED, automated external defibrillator

BLS, basic life support

CA, cardiac arrest

CC, chest compressions

CPR, cardiopulmonary resuscitation

EMS, emergency medical services

N/A, not applicable

OHCA, out of hospital cardiac arrest

This manual defines terms for the manual audit of cardiac arrest calls. Read the instructions carefully. It is important that you the auditor understand what knowledge and information the variables can contribute. Proper coding requires great accuracy and there are many details to be observed. Therefore, it is recommended that you encode a few calls for practice purposes before registering in the database. If for any reason you leave a box blank, enter the reason for this in the comment box at the end (except for variables 15, 22, 24).

**Exclusion criteria for assessment of calls**

- Patient alive at time of call
  - If the patient shows indisputable signs of life, such as attempts to speak or move (fast forward to the end of the audio file to ensure the same patient status, if no cardiac arrest check that no additional calls have been received while waiting for the ambulance)
  - If the OHCA is witnessed by EMS personnel
- Caller access to assessment of patient
  - No close visual contact with the patient, cannot see and/or touch the patient directly or by leaving the phone
- Call interrupted
  - If the call is interrupted before assessment of the patient is possible, and no recall can be identified
- Missing audio files
- Care provider other than SOS Alarm
- Calls where nurses take over the call in the regions: VGR, Skåne and Dalarna. Log the variables you can until the nurse takes over the call and write in the comments field that SSK has taken over the call. If the nurse in these regions is only listening (the dispatcher handles the call until the end), the call should be included. The first digits of the case number indicate the dispatch central number. The nurse belongs to other caregivers in Falun (5), Gothenburg (19) and Skåne (20).

# Call assessment necessary

- 1. Yes, if all inclusion criteria are met.
- 2. No, if one or more exclusion criteria are met.

# Audio file available

- 1. Yes.
- 2. No.

# Country

- 1. Sweden.

# Data collector initials

- Initials of the person conducting the manual audit.

# Filename

- The individual file name assigned to each case from the SOS Alarm “ärendenummer”.
  Example: 12:1234567:1.

# Time of dispatcher addressing caller (MM: SS)

- Is set to 00:00 in all cases.

# CPR started by caller or bystander?

- 1. Yes - if CPR is known to have been started by lay or trained bystanders on the scene before dispatcher starts instructions for CPR.
- 2. No - If CPR is not started by lay or trained bystanders on the scene before dispatcher starts instructions for CPR.
- 3. Unknown.

**Definition:**

— CPR is “already in progress” when callers indicate that they or other lay or trained rescuers on scene have started CPR before the dispatcher starts instructions for CPR.

**Description:**

— Calls where CPR is already in progress should be excluded when calculating the proportion of cases where dispatchers recognize the need for CPR, start CPR instructions, and achieve the first bystander compression. They should also be excluded when calculating the median or average time to these events from the start of the call.

**Instructions for Coding:**

— If CPR is known to start before the dispatcher starts instructions for CPR, mark 1 = “Yes” under “CPR already in progress?”

— If CPR does not start before a call-taker or dispatcher starts instructions for CPR, mark 2 = "No“.

— If it is not known whether CPR started before a call-taker or dispatcher started instructions for CPR, mark 3 = “Unknown“.

— If “No” or “Unknown” is chosen, continue evaluating the call and completing the form.

# Consciousness addressed?

- 1. Yes.
- 2. No.
- 3. Unknown.

**Instructions for Coding:**

1: If signs of consciousness are addressed by either dispatcher or caller (” awake”“, conscious”“, contact”“, reactions” or similar terms). If the caller says that the patient is in cardiac arrest or is dead. Implications, such as “he is all gone” or “he collapsed” is not enough – it needs to be directly addressed.

2: If consciousness is not addressed.

3: Unknown.

# Patient is conscious?

- 1. Yes - If the caller reports that the patient is conscious, is responsive to the caller, or is making purposeful movement.
- 2. No - If the patient can't speak, doesn't respond, or won't wake up.
- 3. Unknown – If information does not emerge.

**Definition:**

— A patient is considered conscious if the caller reports the patient is conscious and/or responsive to the caller. A patient is considered not conscious if the caller reports the patient is not conscious and/or is not responsive to the caller.

**Description:**

— A patient’s level of consciousness is a key indicator of whether he or she is in cardiac arrest. It can be difficult to get a clear answer on whether the patient is conscious. Callers often give contrary answers to this question at different times in the call. CPR instructions should be given when a patient is not conscious and not breathing normally.

| **Examples:**  The caller says her husband is “passed out and not responding“. | Mark 2 = “No”, coding the patient as not conscious. |
| --- | --- |
|  |  |
| The caller does not commit in answering whether the patient is conscious, saying “yes” at one point, “no” at another and “I can’t tell” at another. The dispatcher asks if she can speak with the patient. The caller says, “No, there’s no way he can talk to you“. | If the caller reports that the patient can’t speak, it indicates the patient is most likely not conscious. Mark 2 = “No”, coding the patient as not conscious. |
| A caller says the patient is in a seizure. The seizure then stops, and the caller reports that the patient “is snoring like he’s in a deep sleep and he won’t wake up“. | A patient who “won’t wake up” should be classified as not conscious. Mark 2 = “No”, coding the patient as not conscious. |
| The caller reports the patient wouldn’t wake up a minute ago, but now appears to be “getting better“. The dispatcher tells the caller to shake the patient’s shoulders to see if the patient responds. The caller says he moaned and pushed her arms. | A patient who makes purposeful movement (pushing the caller’s arms away) is demonstrating conscious intent and should be coded as conscious. Mark 1 = “Yes”. |
| If during the conversation, information does not emerge that allows the level of consciousness to be assessed. Select 3 = Unknown. |  |

# Breathing addressed?

- 1.Yes.
- 2. No.
- 3. Unknown.

1: If signs of breathing are addressed by either dispatcher or caller or the caller says that the patient is in cardiac arrest.

2: If breathing is not addressed.

3. Unknown.

# Abnormal breathing addressed

- 1. Yes.
- 2. No.
- 3. Unknown.
- 4. N/A.

1. If signs of abnormal breathing are requested by the dispatcher.

2. If abnormal breathing is not addressed.

3. Unknown.

4. N/A.

- The dispatcher initiates T-CPR instructions without asking about breathing, for example, if the caller says the patient is dead/cardiac arrest.

- On question 10, the caller indicates that the person is not breathing. Not relevant to ask this supplementary question if no breathing has already been identified. Select 4 N/A.

# Patient is breathing normally?

- 1. Yes - If the caller reports that the patient is breathing normally.
- 2. No – If the caller reports that the patient is not breathing or not beathing normally.
  - Descriptions of abnormal breathing includes, but are not limited to, “gasping”, “gasping for air”, “gurgling”, “snoring”, “humming”, “moaning”, “breathing every once in a while”, and “shallow breathing”.
- 3. Unknown.

**Definition:**

A patient is breathing normally if the caller reports the patient is breathing normally. A patient is not breathing normally if the caller reports the patient is not breathing. A patient is not breathing normally if (A) the caller reports abnormal breathing and/or (B) the dispatcher hears abnormal breathing and/or identifies it through the caller’s description of the patient’s breathing. Abnormal breathing is defined as breathing with a rate and/or character different from the victim’s normal breathing at rest.

**Description:**

A patient’s breathing status is a key indicator of whether he or she is in cardiac arrest. It can be difficult to get a clear answer on whether the patient is breathing normally. Callers often give contrary answers to this question at different times in the call. Agonal breathing is very common in cardiac arrest. Callers often use specific words or phrases to describe this kind of breathing. These descriptions include, but are not limited to, “gasping”, “gasping for air”, “gurgling”, “gargling”, “snoring”, “snorting”, “humming”, “moaning”, “groaning”, “breathing every once in a while” and “shallow breathing“. CPR instructions should be given when a patient is deemed not breathing normally and not conscious.

**Instructions for Coding:**

Mark the number for the appropriate answer (“Yes”, No”, or “Unknown”) under “Breathing Normally?”. In cases where callers describe agonal breathing or where the quality assurance rater hears agonal breathing, patients should be coded as not breathing normally.

| **Examples:**  The caller says her husband is drunk and that he keeps “gurgling and gasping for air“. | The descriptors “gurgling and gasping for air” indicate agonal breathing. Even if the caller suspects it’s just because her husband is drunk. Mark 2 = “No, not breathing normally”, coding the patient as not breathing normally. |
| --- | --- |
| The caller says his wife “seems to be breathing okay”, but the quality assurance rater hears a soft snoring sound in the background. The dispatcher does not hear it or hears it but does not identify it as abnormal breathing. | Mark 2 = “No, not breathing normally”, coding the patient as not breathing normally. |
| If during the conversation information does not emerge that allows normal breathing to be assessed. Select 3 = Unknown. |  |

# Did dispatcher recognize need for CPR?

- 1. Yes - If the dispatcher actively orders or passively instructs bystanders to start CPR.
- 2. No - If dispatcher does not order bystanders to start CPR.
- 3. Unknown.

**Definition:**

— A dispatcher or call-taker recognizes the need for CPR when he or she indicates that CPR should be performed during the call.

**Description:**

— The dispatcher recognizes the need for CPR when he or she says any of the following in connection with a response to the victim’s condition: “CPR”, “chest compressions”, “compressions”, “continuous chest compressions”, “CCR”, “rescue breaths”, “rescue breathing”, “ventilations”, or “rescue ventilations“.

**Instructions for Coding:**

— If the dispatcher indicates that he or she recognizes the need for CPR, mark 1 = “Yes” under “Did dispatch recognize the need for CPR?”

— If the dispatcher does not recognize the need for CPR, mark 2 = "No“.

— If the dispatcher does not detect a cardiac arrest, mark 2 = "No".

— If it is not known whether the dispatcher indicated recognition of the need for CPR, mark 3 = “Unknown“.

# Delayed recognition due to deviation from cardiac arrest protocol

- 1. Yes – If cardiac arrest protocol is deviated at any time of the call
- 2. No – Compliance to cardiac arrest protocol
- 3. Unknown

**Instructions for coding:**

— If agonal breathing is misinterpreted and dispatcher instructs caller to put the patient in recovery position at any time of the call, mark 1 = “Yes”

— Deviations due to asking the wrong questions or asking repeated questions despite the question being answered clearly by the caller. If delay due to deviation such as recovery position or repeating questions regarding, e.g. breathing is 60 seconds or more, mark 1 = “Yes” delayed identification of cardiac arrest.

— If the dispatcher does not ask questions so that consciousness and/or breathing can be assessed, mark 1 = "Yes".

— If the dispatcher has asked questions according to the medical support instructions but does not identify the cardiac arrest, mark 2 = "No".

— If recognition is delayed due to circumstances at scene, mark 2 = “No”.

# Time of recognition (MM:SS)

- Enter with numbers, minutes:seconds.
- If CPR is already in progress, mark ”0”.
- If the dispatcher does not detect the cardiac arrest during the call, leave the field blank.

**Definition:**

— The time dispatcher recognizes the need for CPR is the time elapsed from the start of the call to the moment when the dispatcher or call-taker verbally indicates that he or she realizes CPR should be performed.

**Description:**

— Need for CPR is not recognized if instructions for CPR are mentioned by the dispatcher, and he/she then instructs bystander to check the patients breathing first. In this case, time of recognition is when the first compression is made by a bystander.

— Dispatcher and call-taker recognition for the need of CPR is the first of three key time intervals in the provision of pre-arrival CPR instructions.

— Dispatchers and call-takers indicate their recognition when they say any of the following in connection with a response to the patient’s condition: “Cardiopulmonary Resuscitation”, “CPR”, “chest compressions”, “compressions”, “continuous chest compressions”, “Hands-Only CPR”, “CCR”, “rescue breaths”, “rescue breathing”, “ventilations”, or “rescue ventilations“. In some cases, the dispatcher might not say any of these but indicates recognition by starting CPR instructions. In such cases, the time to dispatch recognition of the need for CPR and the time to start of CPR instructions are the same.

— If the dispatcher or call-taker indicates his or her recognition, but subsequently instructs the caller or rescuer either to “lift the patient’s chin and tilt his or her head back” and/or “to look, listen and feel for breathing”, the time to dispatch recognition of the need for CPR should be defined as the moment the dispatcher or call-taker indicates his or her recognition after instructing the caller or rescuer to perform this breathing assessment.

**Instructions for Coding:**

— Enter in minutes (MM) and seconds (SS) the elapsed time from the start of the call to the moment the dispatcher verbally recognizes the need for CPR.

| **Examples:** The dispatcher says, “We need to start CPR right away“. | Enter the time elapsed to the moment when the dispatcher says “CPR“. |
| --- | --- |
| The dispatcher says, “We need to start CPR” at 1 minute and 27 seconds into the call. She then instructs the caller to lift the patient’s chin, tilt his head back and to look, listen and feel for breathing. The caller performs this procedure. It takes 25 seconds, and at 1:52 the dispatcher says, “OK, let’s start compressions“. | Enter 1:52 as the time to dispatch recognition of the need for CPR. |

# BLS competence addressed?

- 1. Yes - If bystander's BLS competence is addressed by dispatcher.
- 2. No - If bystander's BLS competence is not addressed by dispatcher.
- 4. N/A.
- 88. If CA is not recognized.

1: If previous experience, skills or received training is mentioned.

2: If BLS competence is not addressed.

4: Patient is awake.

88: If OHCA is not recognized.

# 16.1 Is the cardiac arrest witnessed?

- 1. Yes - If the collapse is indicated to have been seen or heard.
- 2. No – If the patient is found and there is nothing indicating the time of collapse.
- 3. Unknown.
- 4. N/A.

**Instructions for coding:**

**—** If a patient is found alone and there are obvious circumstances making the time of collapse impossible to interpret, mark 2 = ”No”.

— If the collapse is seen or heard, or if the patient is found after a short time period, mark 1 = ”Yes”.

— If the dispatcher tries to verify if the patient is dead (for example by asking if the patient is warm) and it´s judged that the OHCA just occurred, mark 1 = ”Yes”.

— If OHCA is not recognized, mark 4 = N/A.

# 16.2 Bystanders have previous CPR training, before T-CPR instructions are given?

- 1. Yes.
- 2. No.
- 3. Unknown.
- 4. N/A.

**Instructions for coding:**

— If the caller says yes, mark 1 = ”Yes”.

— If in doubt, for example ”No it was a long time ago” or ”No I don’t remember”, it still indicates that the caller is familiar with the concept of CPR and should be coded as 1 = ”Yes”.

— If the caller says NO I don’t know CPR, mark 2 = ”No”.

— If BLS competence (question 16.0) hasn’t been addressed by the dispatcher, mark 3 = Unknown.

— If OHCA is not recognized, mark 4 = N/A.

Note:

- Question 16.0 describes whether the dispatcher asks the question of BLS competence (evaluates the dispatchers interview).

- If initially there is no one on scene who known CPR, but later someone comes to the scene who knows CPR (after T-CPR instructions are initiated), mark 2 = "No".

# 16.3 Age above 18 years?

- 1. Yes.
- 2. No.
- 3. Unknown.
- 4. N/A.

# 16.4 Probable cause of OHCA is of medical origin and not in combination with trauma, drowning, suffocation, intoxication or apparent pregnancy?

- 1. Yes.
- 2. No.
- 3. Trauma.
- 4. Drowning.
- 5. Suffocation.
- 6. Intoxication.
- 7. Pregnancy.

**Instructions for coding:**

If there is a clear cause to the cardiac arrest such as trauma, drowning, foreign body airway obstruction, poisoning or if the victim is pregnant, answer no. If none of the above, always answer YES, i.e., if it is unclear what caused the cardiac arrest, answer YES.

# 17. CPR instructions started?

- 1. Yes - Instructions start when dispatcher tells the bystander to “kneel by the patient’s side” or similar phrases.
- 2. No - If instructions are not started. Dispatcher instructing to get the patient to a hard, flat surface is not considered CPR instructions.
- 4. N/A.

**Definition:**

— CPR instructions are directions dispatchers provide to guide callers through the process of performing CPR, whether compression-only or conventional CPR (CPR with rescue breathing). Instructions are considered “started” if they are simply started, even if they are not finished.

**Instructions for Coding:**

— If CPR instructions are started, mark 1 = “Yes” under “CPR instructions started?”

— If CPR instructions are not started, mark 2 = "No“.

— If the dispatcher does not detect a cardiac arrest, mark 4 = N/A.

| **Examples:**  A caller is ready to start CPR. The dispatcher begins instructions, saying, “Kneel by the patient’s side”, but the caller stops him abruptly, saying the patient is “waking up and is conscious now“. The dispatcher does not continue the CPR instructions he started.  Caller starts CPR without instructions from the dispatcher and no instructions are provided by the dispatcher throughout the call.  Caller start CPR without instructions from dispatcher, but dispatcher gives instructions after CPR is started. | Code as “Yes”. Although CPR instructions were stopped just after they were started in this example, they were still started.  Coded as “No”.  Coded as “Yes”. |
| --- | --- |

# 18. Is the dispatcher assertive urging to start CPR or passive and ask if the caller wants to start CPR?

- 1. Assertive.
- 2. Passive.
- 4. N/A.

**Instructions for Coding:**

— Coded as “Assertive” if dispatcher tells caller what to do, e.g. “We need to start CPR” or “I need you to start CPR".

— Coded as “Passive” if dispatcher ask caller if he/she wants to do CPR instead of telling caller "We need to start CPR".

— If the dispatcher does not detect a cardiac arrest, mark 4 = N/A.

# 19. Does the dispatcher check the CPR quality

- 1. Yes.
- 2. No.
- 4. N/A.

**Instructions for Coding:**

—Coded as “Yes” if dispatcher is checking quality of CPR. For example, “please count out loud with me”, “are you pushing deep enough? “ “push a bit faster/slower”.

— If the dispatcher does not detect a cardiac arrest, mark 4 = N/A.

— If CPR is not started during the call, mark 4 = N/A.

# 20. Continuous encouraging/motivating techniques in use

- 1. Yes, if encouraging or motivating techniques are in use.
- 2. No, if no encouraging or motivating techniques are in use.
- 4. N/A.

**Instructions for Coding:**

—Coded as Yes, if encouraging or motivating techniques are in use, e.g. “keep on going”, “you’re doing a great job”, “the ambulance is on its way”.

—Encouraging or motivating techniques must be ongoing throughout the call, it is not enough to just say “keep on going” once.

— If the dispatcher does not detect a cardiac arrest, mark 4 = N/A.

— If CPR is not started during the call, mark 4 = N/A.

# 21. Type of CPR instruction

- 1. 30:2.
- 2. CC, continuous compressions (ventilations are not provided).
- 4. N/A.

**Description:**

— This variable describes the dispatcher’s CPR instruction to the caller, i.e. not what is performed on site.

**Instructions for Coding:**

—If dispatcher starts instructions for compressions only, but changes to include ventilations after a while, code as “30:2”.

— If the dispatcher does not detect a cardiac arrest, mark 4 = N/A.

— If CPR is not started during the call, mark 4 = N/A.

— If the dispatcher does not give any instructions, mark 4 = N/A.

# 22. Time chest compression instructions started (MM:SS)

- Enter with numbers, minutes:seconds.
- 99 = unknown.
- If no instructions are started, leave blank.

**Definition:**

— This is the time elapsed from the start of the call to the moment when the dispatcher starts CPR instructions.

**Description:**

— The time at which a dispatcher starts CPR instructions is the second key time interval in the provision of CPR. Instructions to get a patient to a hard, flat surface should not be considered the start of CPR instructions. Instructions begin when a dispatcher tells the rescuer to “kneel by the patient’s side”.

**Instructions for Coding:**

— Enter in minutes (MM) and seconds (SS) the elapsed time from the start of the call to the moment the dispatcher starts CPR instructions.

| **Examples:**  The caller reports that she is ready to start CPR. The dispatcher says, “kneel by his side and put the palm of one hand in the centre of his chest”, at 2 minutes and 12 seconds. | Enter 2:12 as the time at which the dispatcher began instructions for CPR. |
| --- | --- |

# 23. Chest compressions started?

- 1. Yes - If there is a clear sound of compression, if bystander counts loudly or actively say that they are doing compressions or if they say someone else is doing chest compressions.
- 2. No - If none of the above occurred.
- 3. Unknown.

**Definition:**

— Chest compressions are considered “started” if a rescuer does *any* chest compressions, even if the rescuer stops just after starting.

**Description:**

— Determining whether chest compressions are started can be difficult in a minority of cases. Rescuers don’t always count out their compressions, and sometimes their voices or the compressions themselves are inaudible.

**Instructions for Coding:**

— If chest compressions were started, mark 1 = “Yes” under “Chest Compressions Started?”

— If chest compressions were not started, mark 2 = "No“.

— If the dispatcher does not detect a cardiac arrest, mark 2 = "No“.

— If it is not known whether chest compressions were started, mark 3 = “Unknown“.

| **Examples:**  Caller states he will start CPR, puts down the phone and dispatcher is not able to get in touch with caller. | Coded as “Yes”, chest compressions started. |
| --- | --- |

# 24. Time of first chest compression (MM:SS)

- Enter with numbers, minutes:seconds.
- 0 = If CPR started before call.
- If no chest compressions were given during the call, leave the field blank.
- 99 = unknown (when it is not possible to assign a timepoint when chest compressions started).

**Definition:**

— This is the time elapsed from the start of the call to the moment when the caller or rescuer delivers the first chest compression.

**Description:**

— The time to first chest compression is the third of three key time intervals in T-CPR. The time is noted when the first compression is audible, or the caller/rescuer indicates he or she has started compressions (i.e. by counting with dispatcher).

**Instructions for Coding:**

— Enter in minutes (MM) and seconds (SS) the elapsed time from the start of the call to the moment the caller or rescuer delivers the first chest compression. There are often calls in which the time to first compression must be carefully inferred or entered as “Unknown“.

| **Examples:**  The dispatcher finishes instructions for starting compressions, and the caller clearly counts out the first compression at 3 minutes and 23 seconds into the call. | Enter the time elapsed to first compression as 3:23. |
| --- | --- |
| The dispatcher finishes instructions for CPR at 2 minutes and 50 seconds into the call and tells the caller to count the compressions out loud. The caller doesn’t count, however, and, eight seconds later, at 2:58, the dispatcher asks, “Are you doing the compressions?” The caller says, “Yes“. The dispatcher then reminds the caller to count out loud, and the caller begins: “1, 2, 3 …” | In this scenario, it becomes clear that the caller is doing CPR at 2:58 seconds (the caller says, “Yes” when asked if he’s doing compressions.) The dispatcher told him to count out loud at 2:50. Since 8 seconds later the caller said he had been doing compressions, it can be reasonably inferred that the first compression occurred somewhere between 2:51 and 2:55. In the absence of more perfect information, enter the elapsed time as 2:53, the midpoint between 2:51 and 2:55. |
| The dispatcher finishes instructions for CPR and tells the caller to count out loud at 1:46. The caller doesn’t count, but the first of a string of audible compressions occurs at 1:49. | Enter the time elapsed to first compression as 1:49. |

# 25. Was an AED addressed

- 1. Yes - If an AED or similar wording referring to an AED is mentioned by either caller or dispatcher.
- 2. No - If an AED or similar is not mentioned.
- 4. N/A – If cardiac arrest is not detected.

# 26. Was a public AED connected to the patient?

- 1. Yes - If the caller actively says that he/she or someone else has connected the AED to the patient, or it is clearly heard that the AED is on and gives instructions to the bystander. Public AEDs are AEDs that have not been transported to the scene by an ambulance or emergency services. An AED picked up by security guards in a shopping centre or by SMS lifesavers is considered a public AED.
- 2. No - If none of the above occurred, or if police/fire brigade arrived and connected their AED.
- 3. Unknown.
- 4. N/A.

# 27. Did the AED deliver a shock to the patient?

- 1. Yes - If the caller actively says that the AED has delivered shock/shocks, or it is clearly heard that the AED is giving instructions to deliver a shock.
- 2. No - If none of the above occurred.
- 3. Unknown.
- 4. N/A - If you answered no or N/A to number 26.

# 28. Barriers delaying or hindering CPR?

- 1. Hang up phone - When the caller disconnects from the dispatcher processing the call.
- 2. Language barrier - When the caller and dispatcher do not speak the same language and therefore can't communicate effectively.
- 3. Caller left phone - When the caller leaves the phone for purposes other than rendering aid to the patient after receiving instructions to do so from the dispatcher.
- 4. Caller not with patient - When the caller is at a location that prohibits the caller physically assessing the patient.
- 5. Overly distraught - When a caller’s highly distressed emotional state delays or prevents him or her from taking CPR instructions and/or performing CPR.
- 6. Caller refused - When caller refuses for reasons other than a physical inability to perform CPR.
- 7. Caller couldn't move patient - When a caller reports his or her inability to move the patient from an unsuitable location for CPR (e.g. toilet or bed).
- 8. Patient's status changes - When a patient initially thought to be in cardiac arrest presents an indication that he or she is not in cardiac arrest.
- 9. Patient obviously dead - Caller conveys that patient is deceased. Caller provides enough evidence to the dispatcher in support of that conclusion (e.g. rigor mortis, mottled skin, decomposition, foul odour).
- 10. Other - Any barrier apart from those defined above that prevents the start of CPR instructions and/or bystander chest compressions.
- 11. No barrier.
- 12. Unknown.
- 13. N/A (when CPR is not started).
- 14. Does not want to perform rescue breath despite instructions.
- 15. Wants to perform rescue breaths despite instructions not to do them.

**Description:**

— Barriers to CPR are important to track because the recurrence of given barriers can point the way to protocol changes addressing high-frequency obstacles. For example, a common barrier is that rescuers can’t move a patient from a bed to a suitable location where compressions could be effective. Knowing this, managers and medical directors can experiment with protocol language and procedures to help rescuers solve this problem.

**Instructions for Coding:**

— Register all the barriers present in the call that hinder or delay CPR according to the definitions above. Multiple barriers may be present, separate each number with a (,)

| **Examples:**  The caller, a native Spanish speaker, speaks and understands English poorly. The dispatcher knows little Spanish but is able to get the caller to do CPR after several minutes of trying to clarify his instructions. | Code as a delay to start of CPR resulting from “Language barrier”. |
| --- | --- |
| The dispatcher tries to calm a hysterical caller, but the caller screams and then leaves the phone. The caller is heard screaming in the background until EMTs arrive. | Code as “Overly distraught” and “Caller left phone”. |
| A dispatcher tells the caller that she needs to start CPR and that he will help her. The caller refuses, however, saying she has hurt her back and that there is no way she can get the patient from the bed to the floor. | Code as “Other” (and what that “other” barrier was: physical inability). The caller has refused to take CPR instructions but for reasons owing to a physical inability to perform (her bad back). Code as “Couldn’t move patient”. |
| The patient appears to be unconscious in the back yard, but the caller is on a landline phone on the second floor of the house. The caller is thus not able to physically assess the patient’s status. | Code as “Caller not with patient”. |
| The caller reports that the patient is not conscious and not breathing normally. The dispatcher starts instructions for CPR, but the patient opens his eyes and begins to mumble and deliberately starts rubbing his head. The dispatcher recognizes the patient is conscious and discontinues CPR instructions. | Code of “Patient status change”. |
| The caller indicates that the patient is not conscious and not breathing normally. The dispatcher starts instructions for CPR, but the caller subsequently says the patient is “blue, cold and stiff as a board“. The dispatcher discontinues CPR instructions. | Code as “Obviously dead”. |

# 29. Was the event witnessed?

- 1. Yes - If the caller actively says that he/she or someone else saw the patient collapse, or if the caller or someone else heard the patient collapse and were by their side within seconds.
- 2. No - If none of the above occurred.
- 3. Unknown.

# 30. Call continued until EMS arrival

- 1. Yes - If it is addressed or clearly heard that the EMS is by the patient’s side.
- 2. No - If the call is terminated before the EMS arrives at the side of the patient, or if the dispatcher sees that the EMS has indicated “arrived” and ends the call, or the sirens is heard in the recording and call is ended.
- 3. Unknown.

# 31. Caller gender

- 1. Female.
- 2. Male.
- 3. Unknown.

# 32. Caller relation to patient

- 1. Known patient - If caller is family (mother, father, grandparents, uncle, aunt, cousin etc.), in-laws, friends, colleagues, home care staff, neighbour etc.
- 2. Unknown patient – Caller does not know the patient prior to OHCA incident.
- 3. Unknown.

# 33. Health care professional

- 1. Yes - If caller or other on site has health care education/background regardless of level (MD, nurse, paramedic, emergency medical technician, social and health service helper/assistant, social worker at institutions).
- 2. No.
- 3. Unknown.

# 34. Was caller alone at time of call?

- 1. Yes – If no one else can be heard.
- 2. No - If someone else is heard or communicates, indicating that the caller is not alone on site.
- 3. Unknown.
